# Photography

**Published:** 1910-06-04

**Authors:** J. Gray Duncanson


### The Practitioner's Relaxations.

### PHOTOGRAPHY.

By J. GRAY DUNCANSON, M.B., C.M.

I know of no more delectable art than that of photography, and none which can be more useful and profitable to a medical man, and that in so many different ways. It is the most perfect recording method, be it for a microscopic object, a post-mortem specimen, or an interesting and unusual case. When applied to natural history subjects it makes their study the more fascinating, and leads to an intimate and personal knowledge of the ways and means so marvellously developed throughout the animal and vegetable kingdoms. If turned on the face of Nature, the landscape reveals subtleties of beauty never before observed, and endless delight may be interwoven with the country or seaside walk by the selection of pictures as one moves along, even though these may not be actually photographed. This art is no difficult one to attain useful and average ability in: it can be quickly and inexpensively acquired; by using modern methods the time necessary for its accomplishment may be reduced to a minimum and can frequently be snatched in the intervals of ordinary professional work. The better the lens and camera, the more easily the best results can be produced; but it is possible with even the cheapest apparatus to turn out excellent work.

The vexed question of films or plates I will not enter upon, save to say that the latter will, *cæteris paribus*, repay for any extra weight or inconvenience in changing, especially when using the finest anastigmatic lenses at full aperture, by the finer and more uniform negatives obtained.

The following methods apply to either form, though I will speak of plates only for simplicity sake, meaning thereby a gelatine and silver coated sheet of glass.

One of the most interesting parts of photography is to watch the gradual building-up of the properly exposed negative when placed in a correctly compounded developer, but this can be done only in a suitably lighted and necessarily darkened room under conditions of some little discomfort, and much time may be spent in what to some is a useless manner.

My object is to bring to the notice of medical men specially, who have so little time as a rule to spare, a method which I have employed exclusively for the last three years, producing thereby much better negatives than formerly, and by the enormous saving of time enabling me to enjoy photography when otherwise I would have been compelled to abandon it altogether; the method is also absolutely scientifically correct.

I am speaking of tank development. Tanks of many types are upon the market, but the one I use is the "Standa," which has features quite its own and of special value. It consists of an outer receptacle for the dilute developer and an inner light-tight carrier, into which the plates, after exposure, can be easily placed in absolute darkness, and all the other processes can be carried out in bright sunshine or in an ordinary room. Before we can obtain a negative, exposure of the plate in a camera must take place so that the properly-focussed image may fall upon the sensitive surface of the plate, and remain there for a longer or shorter period, according to the intensity of the light and certain other conditions which will be explained later. Now the judgment of correct exposure constitutes the crux of the whole question of successful photography, whatever method of development is afterwards carried out. No man can accurately judge by the eye the actinic value of the light, which is constantly varying every hour, even when the sun is shining brightly on a fine summer's day. I hold most strongly that the possession of an exposure meter is a *sine quâ non*. The investment of half a crown in a Watkins' Bee meter will be saved over and over again in plates alone which would otherwise be spoilt in faulty exposure. This meter is small, the size of a lady's watch; in the face there is an opening, half of which is occupied by a standard tint; the other half reveals a portion of specially sensitised paper, so prepared that when exposed to light it will darken to match the tint, in a given space of time, which is in direct ratio to the actinic value of the light. In the full light of June this takes two seconds; in winter it may require 30 seconds or longer. The speeds of nearly all plates are given on a card supplied with each meter, and having decided the stop to be used, one movement of the dial gives the reading for the correct exposure for contrast gamma. Increase the exposure for very near or dark objects, and diminish it for clouds, seascapes, and open landscape studies. The light actually falling on the subject should be that tested; in the shadows if detail be required there.

Plates have different speeds, and these are usually marked upon the box; but as there are different standards it is as well at first to hold to those given by Watkins, and by adopting a uniform method perfect results will soon be obtained. I am no believer in a very slow plate, but would advise one designated special rapid, and preferably of the orthochromatic variety, and, above all things, the worker for many months should absolutely adhere to one make and speed of plate. Having correctly exposed the plate, it must be transferred, preferably in darkness, or otherwise in a safe light, to the carrier of the tank, and then development has to be carried out. The particles of silver bromide in the emulsion, which have been acted upon by light, are by a reducing agent converted into silver; this action takes place in direct ratio to the light, and in this way the negative is formed.

There are numerous developers on the market. I use Rodinal almost exclusively for plates, and for these reasons: it keeps well in a concentrated solution, works cleanly, and gives negatives of great perfection; it can be adapted to all plates and every class of subject. Fill the tank with a strength of

1-100, take the exact temperature of the solution, which should not be under 55° F. nor over 70° F., and then the following points should be noted:—

1. *Plate*.—Some plates take longer than others, the ratio being about 1 to 5; either this can be determined by experiment or reference again to the Watkins' card.

2. *Temperature*.—The co-efficient for Rodinal is 1.9, so that if 15 minutes at 70° F. gives the contrast desired, at 55° F. you will require to develop for 22.30 minutes.

3. *Subject*.—Portraits require shorter development than landscapes.

Then the following rules always hold good in tank development, and should be rigidly adhered to:—

1. Increased exposure gives greater density of resulting negative.

2. Prolonged development gives more contrast.

These are within certain extreme limits fundamental truths. To make everything clear I will conclude with one example:—

| | | |
|---|---|---|
| Meter time | = 8 seconds | Exposure will be ⅛ sec. |
| Stop | = F 11 | |
| Plate speed | = 130 Watkin's | |
| Developer | = Rodinal 1-100 | Development time 23 min. |
| Temperature | = 65° F. | |
| Subject | = Medium contrast | |

Empty away the developer, replace with clean water for a minute, and then refill with hyposulphite of soda solution, 4 oz. in 20 oz. of water; give about 15 minutes in this and wash the plates for one hour in running water. The tank can be placed under the tap, and with a rubber tube syphon to drain it; the plates will be thoroughly washed in that time. Stand the plates in a rack to dry.

